# ‘It's quite a balancing act’: A qualitative study of parents' experiences and information needs related to the COVID‐19 pandemic

**DOI:** 10.1111/hex.13994

**Published:** 2024-02-22

**Authors:** Lisa Hartling, Sarah A. Elliott, Kelsey S. Wright, Lisa Knisley, Shannon D. Scott

**Affiliations:** ^1^ Alberta Research Centre for Health Evidence, Department of Pediatrics University of Alberta Edmonton Alberta Canada; ^2^ Cochrane Child Health, Department of Pediatrics University of Alberta Edmonton Alberta Canada; ^3^ Children's Hospital Research Institute of Manitoba Winnipeg Manitoba Canada; ^4^ Faculty of Nursing University of Alberta Edmonton Alberta Canada

**Keywords:** COVID‐19, experiences, information needs, knowledge mobilisation, parents, pandemic, qualitative research

## Abstract

**Introduction:**

Throughout the coronavirus disease 2019 (COVID‐19) pandemic, parents and children faced significant challenges as a result of prevention measures implemented to control the spread of the disease. Ensuring that families have access to essential health information is critical for improving health outcomes and adherence to public health recommendations. Understanding parents' experiences and information needs related to the pandemic and associated health measures (e.g., vaccination, mask wearing, social distancing, etc.) will inform the development and dissemination of resources tailored to parents' needs to support informed decision making.

**Methods:**

We conducted a qualitative descriptive study. Between September and November 2021, parents across Canada were recruited online via social media and community organisation newsletters and listservs to participate in focus groups via Zoom. Focus groups were audio‐recorded and transcribed verbatim. Data were coded and analysed using thematic analysis. Participants completed a demographic questionnaire before the focus groups (via SimpleSurveys).

**Results:**

Sixty‐seven parents participated in 12 focus groups between October and November 2021. In relation to experiences, parents felt they were (1) constantly trying to balance everything, and (2) trying to do their best with the information they had at the time when making decisions. Regarding information needs, parents reflected on (1) how difficult it was navigating copious amounts of changing information and finding credible sources to rely on, (2) the need for resources that were easily accessible, credible and in plain language and (3) the need for resources that were tailored to their needs to support them and their children make informed decisions.

**Conclusions:**

Trying to mitigate the risk of COVID‐19 infection and adhere to public health recommendations, while balancing various factors (work, online learning, and social interactions) and navigating changing information, was overwhelming for many parents. Reflecting on their needs, parents suggested tailored resources that provided concise, credible information in plain language to help them make informed decisions and navigate conflicting information. These findings reveal important knowledge gaps and highlight areas that need to be addressed to support parents during the pandemic period and beyond.

**Patient or Public Contribution:**

Members of our established Paediatric Parent Advisory Group (P‐PAG) were involved as collaborators throughout the planning (grant proposal), development and execution of the study. P‐PAG members gave input on the design of the questionnaire, interview guide, recruitment strategy and interpretation of findings.

## 1 INTRODUCTION

Since March 2020, the world has been grappling with an unprecedented pandemic caused by a novel coronavirus disease 2019 (COVID‐19). The COVID‐19 pandemic impacted the daily lives of families worldwide, and many faced significant lifestyle stressors as a result of varying public health and safety measures. Throughout the pandemic, parents had to navigate those stressors within the evolving and changing pandemic landscape. Globally, parents described stress in their households while navigating the health needs of their family members.^1^ Across Canada, disruptions in activities, school, employment and support as a result of changes in public health mandates and evolving health recommendations presented unique stressors for parents.^2^ In the absence of a vaccine, the primary approach to slowing the spread of the disease was to encourage social distancing and staying at home.^3^, ^4^, ^5^ In March 2020, recommendations for work‐from‐home policies were issued, and school closures were in place until May 2021. Many schools across Canada were closed for up to 27 weeks, during which time students of all levels were expected to learn virtually. Mask mandates were introduced across the provinces from June to November 2020, and in December 2020, Canada began administering COVID‐19 vaccines to adults. Canada approved and began administering COVID‐19 vaccines to adolescents between the ages of 12–17 years in May 2021, and children 5–12 years in November 2021. In June 2022, vaccines for children aged 6 months to 5 years became available.

Parents frequently faced challenges related to adherence with public health measures such as masking and social distancing,^6^ vaccinations^7^ and a wide range of social interactions.^8^ Parents were constantly needing to navigate information that was in flux and often confusing, from innumerable sources to make decisions for their families.^9^, ^10^ The abundance of health information did not effectively serve parents or decrease their uncertainties.^11^ The use of social media by public health agencies and decision‐makers to disseminate information and encourage uptake of public health messages did not always follow best practices for health communication^12^ (e.g., transparency, promptness, clarity, engagement).^13^ A lack of trust in decision‐makers and their messages can influence the public's ability to hear critical messages and align their behaviour accordingly.^14^ This, coupled with scientific uncertainty, may have led to varying adoption and divergent views of protective measures, such as vaccines and masking.

Previous research has shown parents need tailored, relevant information to support them in making healthcare decisions for their children and families.^11^, ^15^, ^16^ Rather than adding to the sheer volume of information, specifically tailored knowledge mobilisation^17^ products (sometimes referred to as knowledge translation tools, e.g., plain language videos or infographics, that are intended to move evidence into action^18^) can help families navigate conflicting information, identify relevant information and facilitate information uptake.^19^, ^20^ Knowledge mobilisation encompasses a range of activities related to the production and use of research.^21^ Connecting end‐users to research evidence has the power to improve patient knowledge, inform health decision‐making and optimise health outcomes.^18^ For effective health messaging for parents, researchers first need to understand their specific experiences and information needs related to the COVID‐19 pandemic. Through targeted knowledge mobilisation efforts (curating lay‐friendly resources and sharing them where they look for information), parents' concerns and questions about COVID‐19, as well as health issues that may persist beyond the pandemic, may be addressed more effectively.

Our aim was to understand parents' experiences and information needs related to the COVID‐19 pandemic and associated public health measures (e.g., vaccination, mask wearing, social distancing) to inform the development and dissemination of knowledge mobilisation resources tailored to parents' needs. The pandemic highlighted a pervasive challenge arising from the public's lack of confidence in science and government authorities, and the crucial need for widespread dissemination of clear, transparent and research‐based information.^22^, ^23^ Ensuring that Canadian families have access to essential health information is critical for improving health outcomes and adherence to public health recommendations, both during and beyond public health emergencies.

## 2 METHODS

In this exploratory study, we used a qualitative descriptive approach to understand parents' experiences and information needs.^24^ Recruitment and data collection for this study occurred from September to November 2021 (Canada's fourth and fifth waves of COVID‐19 infection).

### 2.1 Recruitment

Parents were recruited online between September and November 2021, via social media posts (Twitter, Instagram, Reddit, Facebook) as well as contacting relevant local and national organisations (e.g., Multicultural Health Brokers Cooperative, Refugee Health Coalition, Catholic Social Services, Children's Healthcare Canada, Translating Emergency Knowledge for Kids) to share within their networks via newsletters and electronic mailing lists. Potential participants were directed to an online survey containing the study information letter, acknowledgement of consent and demographic questionnaire. Once potential participants self‐identified as eligible (parents of children <18 years, who spoke English, resided in Canada, had email and access to the internet), they consented by completing the demographic questionnaire. Participants were then approached via email to schedule their focus group session. Focus groups were arranged based on participant availability and conducted during October and November 2021. Given that in‐person data collection methods were not possible during the pandemic, all focus group sessions were conducted using the Zoom video conferencing platform.

### 2.2 Focus groups

Focus group interviews were conducted by K. S. W. (woman, MSc), S. A. E. (woman, PhD) and K. R. (woman, MN), who are trained in qualitative methods. Focus groups followed a semistructured interview guide (Supplementary Information S1: 1). The interview guide followed a format of general to specific; parents were first asked to describe their experiences and information needs related to the pandemic, and then asked specific questions about public health measures (e.g., masking, social distancing, vaccinations, etc.), where they seek information about COVID‐19 and what support and information they felt were needed. This interview guide was pilot‐tested among research team members (some parents) before focus group sessions. The semistructured nature of the interview guide allowed for meaningful engagement with parents, exploring topic areas of particular interest for those in attendance. Upon entry into the Zoom session, participants were asked to provide verbal consent to being recorded. Once participants provided consent, focus group sessions were audio recorded.

### 2.3 Data analysis

Data collection and analysis occurred concurrently. We used thematic analyses as the data analysis technique.^25^ Each focus group session was transcribed verbatim by a third‐party transcription service (Simply Transcription), and cleaned for accuracy and completeness. Transcripts were analysed using an inductive, iterative process, whereby verbatim codes were applied to meaningful participant quotes and then relevant codes were grouped under appropriate titles. Research team members (K. S. W., S. A. E. and L. H.) reviewed and discussed codes and preliminary categories. Code groups from every transcript were grouped into related themes that best depicted major topic areas indicative of participants' experiences and information needs. Preliminary findings and interpretations were continuously reviewed and discussed among the research team. Qualitative data management and analysis were facilitated through NVivo Software (release 1.71; QSR International PTY Ltd.). Quantitative data (participant demographics) were analysed using Microsoft Excel (v. 2016; Microsoft Corporation). Frequency counts were used to describe the study sample.

### 2.4 Rigour

Throughout the focus groups and during analysis, members of the research team took field notes documenting nonverbal communication from participants and personal reflections about the parents' experiences. These field notes allowed the researchers to actively interrogate their biases to better reflect the genuine perspectives of participants during the analysis process.^26^ Verbatim codes from focus group transcripts supported the inductive analysis process, whereby relevant themes and supporting quotes could best represent the unique experiences of parents during the COVID‐19 pandemic. Continual discussions among the research team members promoted critical reflection during the analysis process.^27^ The three facilitators identified as White females. One was a nurse, and two were health researchers. All three have worked with children and families in various health research and clinical contexts. All value the experiences and perspectives of parents and believe it is important to share their viewpoints and information needs related to the pandemic.

### 2.5 Stakeholder engagement

Members of our established Paediatric Parent Advisory Group (P‐PAG)^28^ were involved as collaborators throughout the planning (grant proposal), development and execution of the study. P‐PAG members gave input on the design of the questionnaire, interview guide, recruitment strategy and interpretation of findings.

## 3 RESULTS

Eighty‐seven parents consented to the study and completed the demographic questionnaire. Sixty‐seven of those parents participated in 12 focus groups, with three to seven parents in each group. Focus group sessions ranged from 63 to 90 min in duration.

### 3.1 Demographic information

Demographics collected from 87 participants are displayed in Table 1. Notably, 14% were men, 74% identified as White, 46% lived in the suburbs, 95% were partnered, 75% had two or more children and 13% were born outside Canada. The majority of participants were also highly educated (85% having a postsecondary or graduate degree) and from high‐income households.

**Table 1:** Demographic characteristics of participants (*N* = 87).

	*N* (%)
Which gender do you identify with most?
Man	12 (13.8)
Woman	73 (83.9)
Nonbinary	2 (2.3)
Which of the following race and ethnicity groups best describe you?	
Black (includes African, Afro‐Caribbean, African Canadian descent)	10 (11.5)
East Asian (includes Chinese, Korean, Japanese, Taiwanese descent)	6 (6.9)
Indigenous (includes First Nations, Métis, Inuk/Inuit descent)	2 (2.3)
Middle Eastern or North African (includes Arab, Persian, Afghan, Egyptian, Iranian, Lebanese, Turkish, Kurdish, and other West Asian descent)	2 (2.3)
South Asian (includes East Indian, Pakistani, Bangladeshi, Sri Lankan, Indo‐Caribbean, and other South Asian descent)	2 (2.3)
Southeast Asian (includes Filipino, Vietnamese, Cambodian, Thai, Indonesia, and other Southeast Asian descent)	3 (3.8)
White (includes European descent)	60 (68.6)
Not listed or other	2 (2.3)
If you selected Indigenous, would you describe yourself as Two‐Spirit?
Yes	1 (1.1)
What is your age?	
20–30 years	7 (8.1)
31–40 years	45 (51.7)
41–50 years	34 (39.1)
51 years and older	1 (1.1)
Do you have a supportive adult in your everyday life (e.g., spouse, common‐law partner, other partner/relationship)?	
Yes	83 (95.4)
No	4 (4.6)
What is your yearly household income?	
$25,000–49,999	6 (6.9)
$50,000–74,999	5 (5.7)
$75,000–99,999	11 (12.4)
$100,000–149,999	21 (24.1)
$150,000 and over	34 (39.1)
Prefer not to answer	10 (11.5)
What is your highest level of formal education completed?	
High school diploma	1 (1.1)
Some Postsecondary	4 (4.6)
Postsecondary certificate/diploma	10 (11.5)
Postsecondary degree	35 (40.3)
Graduate degree	37 (42.5)
Where does your household live?	
Inner city	29 (33.3)
Suburb	42 (48.4)
Town	13 (14.9)
Farm/rural	3 (3.4)
How many children do you have?	
1	22 (25.3)
2	43 (49.4)
3^a^	22 (25.3)
Primary language spoken at home^a^	
English	79 (91.0)
French	2 (2.3)
Somali	2 (2.3)
Tamil	1 (1.1)
Arabic	1 (1.1)
Persian	1 (1.1)
Czech	1 (1.1)
Were you born in Canada?	
Yes	76 (87.4)
No	11 (12.6)

^a^ Denotes languages listed by participants.

**Focus group findings**

We organised our findings by phenomena of interest. First, we present parents' experiences followed by their information needs.

### 3.2 Parent experiences

Two major themes emerged from the analysis related to parent experiences. The first overarching theme related to the balancing act parents felt they were doing while (a) managing various factors such as work, online school, family commitments and (b) weighing the risks and benefits for their children regarding health issues. The second theme explores how parents thought they were doing their best with the information they had at any given time, which meant (a) navigating the many unknowns related to the pandemic and (b) having to make decisions with little, changing, or discordant information.

#### 3.2.1 Theme 1: A balancing act

A major theme throughout the focus group discussions was the concept of ‘balance’. Parents relayed their experiences around trying to (a) manage various factors such as work commitments, online learning, changing school schedules, children's social interactions and children's mental health, while (b) trying to mitigate the risk of COVID‐19 infection and adhere to public health recommendations.

##### Subtheme 1: It is stressful trying to manage everything as a parent

Many parents spoke about how stressful and anxiety provoking it was trying to manage as a parent during the pandemic, juggling work commitments with lockdowns and online learning. As one parent stated, ‘It's a lot of balls to be juggling as a parent right now’ (FGD_001_P002).

While some reflected on how lucky they were to continue their job online, managing that with online learning was incredibly stressful, as one parent commented:

I was lucky enough that my job was actually able to be remotely online, so I was home all the time, but having to counteract a child who was doing online learning with another kid who just wanted her older sister's attention was incredibly stressful. And there were definitely moments that I think the trajectory of my career suffered. You know, I had to attend meetings with kids screaming in the background because they couldn't stop fighting. And it was incredibly stressful. (FGD_012_P002)

Parents further reflected:

The beginning of the pandemic and the lockdown was such a stressful time in our house because … we now had a child starting kindergarten online and my husband working online and all of us in this very small space and sometimes they would be sharing the table and each on their different meetings and then there's, like, a three‐year‐old in the background kind of fending for himself and it was just chaos all the time. (FGD_006_P005)

For many families, ‘It was really a situation where all of us had to dig deep … and learn to live together and balance our careers, our schoolwork, and our socialisation needs’ (FGD_012_P002).

##### Subtheme 2: Adhering to public health recommendations

Parents discussed how they had to ‘try and find the balance' between adhering to public health recommendations (e.g., masking, social distancing) to mitigate the risk of infection with their children's socialisation and mental health needs: ‘These have been like probably the most challenging conversations over the course of the last 18 months—is the balance between, like, the risk to health and then the risk to mental health’ (FGD_005_P008). One participant added:

I think as a parent, you're kind of always trying to find this balance between, like, doing enough so that your kid still has like a ‘normal childhood’ but also, you know, working with the restrictions and making sure you keep them safe … (FGD_001_P003)

For many parents with younger children, it was hard to find that balance, ‘… we wash our hands and wear our masks and when she has a runny nose she stays home … trying to balance that with wanting to give them a life still, and she's five. Yeah, it's so hard. It's really hard’ (FGD_002_P007).

Parents of older children found it harder to navigate the balance between socialisation and mental health needs with the risk of COVID‐19 infection:

I find it harder with my older child because he's watching some of his friends get together and so there's that feeling of exclusion, that they're old enough and emotionally mature enough to feel left out. And then it's just difficult to explain that to them, right? (FGD_002_P005)

As one parent said, ‘I just try to make good safe decisions and also balance that out with giving everybody that socialisation and opportunities that they missed for so long’ (FGD_006_P005). Another parent noted, ‘You also want to balance their mental health and you're like “I can't keep them in a bubble”’ (FGD_001_P002).

Parents also suggested that as the pandemic went on, poor mental health became a bigger risk than infection, ‘You know, eventually it became mental health as a bigger risk than COVID‐19 for, you know, for us where we are very isolated’ FGD_005_P012.

#### 3.2.2 Theme 2: Doing their best using the information they have at the time

Parents discussed the challenges they faced when navigating the changing pandemic landscape and making health‐related decisions for their children. The need to make decisions with little or conflicting information was described as stressful and challenging.

##### Subtheme 1: Navigating the unknown

Parents often mentioned the many ‘unknowns’ that were causing a lot of stress and anxiety for them and their families. First, not knowing how long the pandemic would last was something parents struggled with, ‘When is it going to be done? Not that it will ever be done but it's just sort of that unknown if it's going to pop up again’ (FGD_010_P004). Second, parents questioned what the impact of COVID‐19 infection would be on their child's immediate and long‐term health, ‘Between COVID in children and COVID in adults. Does it present the same way? Does it have the same long‐term effects?’ (FGD_003_P001) Third, parents had questions around COVID‐19 variants and the utility of the vaccine. ‘Yeah, I think it's just, there are some unknowns out there, both in terms of, like, will this continue to mutate and change and, or will it be eradicated with vaccine eventually. I don't know’ (FGD_010_P001).

Parents also noted how difficult it was to navigate the many unknowns in general:

The anxiety has been really high. Lots of conversation in our family about things. Lots of unknowns that we really can't, can't answer questions and we just kind of have to leave that unknown there on the table and we can just talk about our feelings around them. (FGD_005_P001)

Navigating these topics and having to make decisions based on the information they had at the time was stressful and difficult for many families, ‘I just think like the whole unknown is like—was super hard for everyone and having to make decisions … like life‐threatening potential decisions’ (FGD_002_P003).

##### Subtheme 2: Decision making

Parents described at length how difficult it was to make any decisions during the pandemic. One parent even said, ‘In COVID, there are no good choices’ (FGD_008_P002), illustrating the hopelessness many others articulated during focus group sessions. Again, the idea of ‘balance’ was prominent, when parents tried to find, ‘The balance between, the risk to health and then the risk to mental health’ (FGD_005_P007). Throughout the conversations, many parents said, ‘You make the best decisions you can with the information you have at the time’ (FGD_001_P006). Although many admitted that was frustrating at times, it seemed like a mantra of sorts that parents would use during difficult decisions, ‘Make the best decision you can on the day of, and just say, “okay, this is what we are going to do”’ (FGD005_P008). One parent noted that as ‘Everybody has a different value set and you make different decisions for your kids based on that’, they stepped back from judging others and recognised that many parents are trying to ‘Do the best you can in the situation that you are in’ (FGD_005_P007).

Parents also spoke about the decision‐making process being different when making decisions about their own versus their child's health. Many parents shared that while they themselves had received COVID‐19 vaccinations, they were wary about getting their children vaccinated, ‘I took the vaccine, yes, but for my child, I think he's still so young to be experimented on’ (FGD_008_P005).

As one parent noted:

It's different making a decision for somebody else rather than making it for yourself … the choice to get vaccinated myself, easy decision … making the choice for my kids is a little different just because I'm making the choice for them. (FGD_003_P001)

Another parent agreed:

Yeah, you don't want to make the wrong choice for your child's body, right. Like I didn't hesitate getting vaccinated like everyone else—not everyone else—but like I didn't hesitate making that decision for myself. But it does feel different when you're making that choice for your child even though we make choices for our children all the time. You know, big, important ones, but yeah, it's a little bit of that unknown. (FGD_003_P002)

Another notable area of discussion was parents' concerns around making decisions that may impact their children's long‐term health and development (e.g., physical consequences of the vaccine on their children's health, pulling them out of extracurricular activities). Further, parents were concerned that their children were missing key periods for skill development (e.g., fine motor skills, gross motor skills) that could impact their future life skills. ‘Have I isolated them so much during this time that now I have negatively impacted their long‐term outcomes, you know, their long‐term developments, their ability to even swim?’ (FGD_003_P003).

### 3.3 Information needs

Many of the parents' experiences related to decision making and navigating the unknown were reflected in their information needs. Three themes captured parents' information needs. First, parents spoke of the challenges they faced navigating the abundance of information, especially sifting through multiple, changing information sources related to public health recommendations (e.g., vaccinations, masking, social distancing) and the need to be able to find credible sources to help them make informed decisions. Second, parents noted the need for easily accessible, plain language and evidence‐based information to help them make decisions. Third, parents spoke about wanting specific resources tailored to their needs (e.g., how to talk to their child about COVID‐19), as well as general resources that may support public health measures in the future (e.g., vaccinations, information about long COVID‐19).

#### 3.3.1 Theme 1: Challenges navigating an abundance of information

A substantial contributor to parents' unease throughout the pandemic was (a) navigating information from various sources that were constantly changing and (b) finding reputable, trustworthy information sources. Parents suggested ways in which resources could be developed to help support them navigate the misinformation circulating during the pandemic.

##### Subtheme 1: Frustrations navigating multiple, changing information sources

Overwhelmingly, parents described their confusion with information constantly changing:

You know, especially at the beginning, the information felt like it was changing a lot of the time … And so, trying to just do a good job of staying up to date with the information that we have as scientists and doctors gained more information … So, yeah, constantly confusing but just trying to sort through that and do your best. (FGD_006_P005)

As well, many mentioned feeling fatigued when sorting through information to find the specific answers they were seeking, ‘… that whole process is exhausting—of trying to find the information, stay on top of the information …’ (FGD_001_P004).

Parents also mentioned their frustration with the lack of transparency about why some public health decisions were made:

When a decision gets made, why is that the decision as opposed to a different decision? What factors were considered, right? And I found sometimes that that was lacking. And I think when you don't justify why certain decisions are made, it breeds misinformation and it plants seeds of doubt… I found that not always was there a really great explanation of the why behind the decision. (FGD_002_P006)

##### Subtheme 2: Difficulty finding information from a credible, reliable source

While parents relied on multiple sources (e.g., newspapers, televisions, radio, social media, and online news websites) to obtain information about the pandemic, all acknowledged the lack of information that appeared trustworthy and the abundance of ‘clickbait’ or misinformation online, ‘There's just a ton of misinformation out there and maybe a lot of huge question marks that just leave me always wondering if I'm doing the right thing or not (FGD_002_P001). Parents specifically criticised social media like Facebook for the harmful misinformation they noticed (especially around vaccines), as one parent commented, ‘I think there's a lot of misinformation out there about like if you're vaccinated but around kids who are not vaccinated, … how does it spread? (FGD_001_P003).

Further, parents highlighted the need to question where they and others were getting information:

You can't just say ‘oh, I saw it on the internet and obviously it's true’ … question where you're getting your information. Is it—are you talking to your doctor about this or you saw a post on Facebook? And is that really where you want to get your information and make your decisions based on? (FGD_004_P004)

The difficulty in finding credible sources of information to help them make informed decisions was also discussed:

It's just very hard to know what to read, who you can trust, and where to get, like, a good source of information … So, it kind of made it all really hard for people to know where to go for good information and feel that it was like accurate and reflected truly what was happening. (FGD_008_P005)

Though many relied on information branded with government or health agency logos, they stressed the need for clear and concise information supported by credible sources:

I really like how [Speaker 7] put it in terms of if we could have the data in sort of plain language, but also from a source that could be seen as unbiased and impartial. I think that would go quite far as well. (FGD_001_P005)

Many parents also suggested resources were needed to help assess the credibility of information, e.g., ‘It would be helpful to have, a cheat sheet, for people looking for information on what's, like, good information or how to tell what sources to look at, depending on what you're interested in’ (FGD_010_P001). Another parent added:

It would be helpful if there was something for parents, like, how to, what is a legitimate source of information … How do you look at an article, you know, like the real simple, yeah, like is it peer‐reviewed? So, when they are looking at information, they can, you know, try to make a decision whether it's a reliable source (FGD_010_P004).

#### 3.3.2 Theme 2: Parents want easily accessible, concise, evidence‐based information to make informed decisions

Parents agreed that they wanted to make informed decisions for their families, ‘Tell me the thing that you found and tell me why, so I can evaluate it for myself on some level’ (FGD_002_P003), but struggled with either the lack of information available or the inaccessibility of information they were given. Another parent added, ‘Like the order is there, “this is what you should do", but the *why* is missing’ (FGD_002_P005).

Parents noted that they wanted the information in a condensed version with links for further information if needed,

Giving us, helping us find the information—or at least giving us a condensed version and then citations as to, like, scientifically held facts and research studies, it would be nice to be able to have a little bit more of a high level of, like, understanding of everything. So, yeah, just having that information and having good citations behind them and just helping me have a better understanding. (FGD_005_P003)

Parents also wanted information presented in plain language:

Most of us don't really know much about this stuff, you know. Not everyone went to school to study nursing or stuff like that. If there is more information about these vaccines, you know, that's not just limited to those who understand the medical literature, complex grammar, and all that, you know, I think it will be useful. (FGD_009_P005)

Another parent commented, ‘You have to make the information accessible …’ (FGD_002_P003).

It was also agreed that there was a need for data to support the messaging:

If we could have something with enough plain language but still … with the data that maybe someone like me could go and dive into. Whereas someone who wouldn't understand all that data, can still get the value from it. (FGD_001_P007)

Other parents then discussed how that information should be shared:

It would be a lot more public campaigns, maybe pamphlets, maybe billboards, something they can have access to in their day‐to‐day life that doesn't require effort on their part [to obtain] and has a clear, concise message, ‘this is the risk that you need to consider when making decisions about how you live your life right now’. (FGD_001_P006)

It was also suggested that:

Shorter videos like ads on T.V. and that type of stuff, that might be more beneficial especially if they're shorter and drilled down because it's easier to spend a minute watching an informative video than a few minutes reading a pamphlet especially if you're in a rush. (FGD_006_P003)

Another parent mentioned how helpful it was when health professionals or scientists:

Break it down into a very simple terms, use lots of graphics, and break down the peer‐reviewed articles into, I guess you would say, layman terms … knowing that that stuff has been studied and peer‐reviewed obviously helps, but if you could do it in a way that makes it accessible, always certainly helps. (FGD_008‐P003)

#### 3.3.3 Theme 3: Parents want tailored resources to support them and their children

When asked about their information needs, parents reflected on (a) the need for resources tailored to their situation as parents navigating the pandemic (e.g., helping answer questions such as, what are the long‐term effects of COVID? What are the long‐term effects of vaccines?), as well as (b) general resources to help with public health campaigns, supports beyond the pandemic.

##### Subtheme 1: Need for tailored resources.

Parents wanted resources to help them talk to their children about the virus as well as the different public health measures to curb the spread of the disease (vaccines, masking, social distancing, etc.), as one parent commented:

I would love to see more resources that speak directly to kids about the virus in an age‐appropriate way, because I think that's something that most parents probably haven't spent too much time thinking about until it all came rushing in at once, how to explain certain behaviours of distancing, or why we can't go to the pool, and things like that. (FGD_011_P001)

This was echoed by another parent who, ‘Would like to see more information that is written for children and for youth about *why* vaccines are important and what the virus is’ (FGD_011_P002).

Parents also noted that resources to explain how vaccines work and presenting information in a neutral way was key to helping them make informed decisions about vaccination.

I want to know, like, which is the safest vaccine … then also the truth on the side effects of these vaccines. Because we understand that, yeah, they might have side effects, but then it's good we be told, like, the real thing so that we know what we are getting ourselves into. (FGD_008_P005)

##### Subtheme 2: Supports for the future

The need for resources to support families in the future, beyond the pandemic was also discussed. These topics related to some of the unintentional consequences of the pandemic (e.g., mental health challenges):

I would *love*, like, scripts or prompts or in age‐appropriate language about how to talk to your kids about these things … the mental stress that they're feeling. What are the prompts but also what are the flags that we should be looking for to know when more help might be needed to handle this. (FGD_004_P003)

As well as how to navigate life postpandemic, ‘You know, having tips provided on how we can mitigate risks and help our kids kind of adapt and get back to, go back into the groove of things after, or if there is a different reality, how to adjust to that, would be nice’ (FGD_005_P007).

Parents also thought it was important to increase awareness about some of the lessons learned during the pandemic such as ‘How important mental health is, and that kids are going to have some different kind of, different fears in the future … it can't be ignored to build a better society moving forward’ (FGD_009_P002).

## 4 DISCUSSION

This study provides a rigorous and insightful analysis of the perspectives of parents related to managing their children and decision‐making regarding their children's health during the pandemic. Throughout focus groups, parents discussed their experiences navigating the pandemic and concerns they had about their children's physical, developmental and mental health and well‐being. A major theme throughout these conversations was how difficult it was for parents to balance everything and make informed decisions, specifically around the challenges (less socialisation, mental health concerns) and benefits (mitigating infection) of adhering to public health measures. Parents also reflected on the need for clear and concise plain language resources that were tailored to their needs and the decisions they needed to make for their families (e.g., vaccinations) as well as resources that supported them navigating various sources to find credible information.

### 4.1 Parent experiences

Parents and caregivers experienced high levels of stress during the pandemic,^29^ which exacerbated existing vulnerability and contributed to uncertainty for many as they juggled employment, home schooling, child care and the need to constantly make decisions to keep their families safe.^30^ Previous studies have described how friendships and relationships were strained due to both limited communication with peers and relatives outside of the household and excessive time together within the household.^31^ Likewise, conflicts in opinions and practices regarding public health measures between family units placed even more strain on delicate family relationships under pressure.^30^, ^32^

Navigating the overwhelming and often conflicting information available at the time to make informed decisions about vaccines, masking, social distancing and so forth was difficult. Similar to recent findings by Kohler et al., parents were frustrated with the lack of clear communication, and how policies seemed to change so frequently regarding public health measures.^33^

Parents expressed feeling overwhelmed with the amount of information available online, particularly regarding COVID‐19 vaccinations and isolation requirements.^7^, ^8^ Though many participants believed available COVID‐19 vaccinations were safe for adults and revealed that they themselves were vaccinated, others questioned vaccine safety with young children and the necessity of getting vaccinated if children had already been infected. This finding aligns with recent reports demonstrating a downturn in childhood vaccinations despite a slight increase in adult vaccination rates.^34^ An interesting phenomenon was also parents' experiences with the difficulty *making a decision* for their children's health in comparison to their own. This has been previously reported with specific reference to the complexity of vaccine decision making.^35^ It also echoes a recent finding in which parents reported that making a decision about vaccinating their child was ‘challenging and expressed difficulty sourcing and evaluating evidence, and balancing their own conceptions of healthcare decisions with societal expectations and political messaging’.^36^

Further, the need to make decisions with little information or conflicting information was stressful and challenging for many parents.^30^ Advancing knowledge and understanding around the situated experiences of parents during the pandemic will inform decisions for implementing knowledge mobilisation strategies as we move beyond the pandemic and for other public health issues, including regular seasonal outbreaks and future epidemics.

### 4.2 Information needs

Similar to what has been previously reported,^33^ we found parents used a variety of sources to obtain information about COVID‐19, and often sought information online (Google search, YouTube, government websites). However, parents mentioned the need for simple, evidence‐based knowledge mobilisation products (easily accessible, preferably online resources) to support their informed decision making. The notion of breaking things down into plain language and the use of visuals to help communicate data was valued by many parents. This aligns well with previous research showing that parents preferred plain language summaries or blog shots over scientific abstracts or online information which used technical terms and medical jargon.^37^, ^38^

Topics parents wanted resources for varied from supporting parents with how to talk to their child about COVID‐19, as well as the various public health measures (e.g., vaccines, masking, social distancing, etc.) to information about long COVID‐19 to help them make informed decisions. Questions arising about the effects of the pandemic on children's long‐term physical and mental health were at the forefront of many parents minds.^39^ Additionally, clear communication around *why* public health recommendations change and their credibility was considered imperative to support parents' trust in public health messaging.^40^, ^41^ Tailored resources for parents, as well as resources for children and youth, to support them in navigating the pandemic may have alleviated some of the anxiety and stress families felt.^34^, ^42^ Initiatives responding to future pandemics or public health emergencies may be well served by working with families to deliver information via trusted sources, tailored to what parents want to know and how they can find it.^43^

Although parents mentioned that they relied on the latest health research for their decision‐making about COVID‐19, several participants outlined their frustration with a lack of trustworthy information sources, or their inability to determine what a credible source is. This finding was not surprising given the phenomenon of global misinformation during the pandemic which made it difficult to find trustworthy reliable sources of information.^44^

### 4.3 Research implications

As experiences and information needs change throughout the pandemic and beyond, it is essential for researchers to effectively engage with parents and caregivers to better meet their information needs.^45^ Knowledge mobilisation products should provide tailored information for parents in usable and relevant ways to best inform an audience already inundated with emotionally charged^2^ and potentially misleading COVID‐19 information.^22^

With this in mind, we worked in partnership with our P‐PAG to develop six knowledge mobilisation resources to help parents navigate their information needs and concerns related to COVID‐19 and the pandemic. The tools (videos and interactive infographics) provide links to credible sources of information and address issues raised by parents regarding navigating their child's social world, vaccination, and parenting when your child has COVID‐19, and are freely available at https://www.echokt.ca/tools/covid/. These resources are based on our findings, as well as those from another qualitative study exploring parents' experiences managing children with COVID‐19.^46^

## 5 LIMITATIONS

Although our team worked with groups who supported diverse cultural communities (e.g., Multicultural Health Brokers Cooperative, Refugee Health Coalition) to ensure variation in our sample, the demographic attributes highlight a lack of diversity across parent participants. It was also noted by parents throughout the focus groups that they were coming from ‘a place of privilege’ and it would be beneficial to hear from those less advantaged parents, newcomers to Canada or those whose first language was not English. We recognise that vulnerabilities of systemically oppressed populations were exacerbated during the COVID‐19 pandemic, including substantial barriers to healthcare access^47^ and that our findings represent a select cohort of parents across Canada. Additionally, although 87 parents completed the online demographic questionnaire, only 67 completed the focus group sessions. There may be population groups like fathers, single parents, those with one child and those born outside of Canada not represented in the study sample.

Participants were required to speak English, and since recruitment and data collection occurred online (as all in‐person research activities were suspended during the pandemic), our sample only included participants with access to technology. As a result, our findings may not fully reflect the experiences and information needs across different parent groups, and generalisability is limited.

Recruitment and data collection for this study occurred from September to November 2021 (fourth and fifth waves of infection), which represented a distinct period of COVID‐19‐related experiences. While this could be considered a strength (capturing experiences ‘in the moment’), the rapidly changing nature of the COVID‐19 pandemic likely produced evolving concerns for parents since these focus groups occurred. Many concerns, like the long‐term effects of COVID‐19 and the pandemic on physical and mental health of children, will be relevant for parents and families for some time. Further, information gained through this and similar studies is important for effective messaging regarding public health measures in general (e.g., routine vaccinations) as well as planning for future epidemics and outbreaks.

## 6 CONCLUSIONS

Parent experiences and information needs during the COVID‐19 pandemic reveal important knowledge gaps and highlight areas that need to be addressed to support parents during the pandemic period and beyond. Parents reflected feeling overwhelmed trying to balance work, life and making health‐related decisions for their children while navigating the changing pandemic landscape. Parents want information that directly relates to the decisions they need to make for their children (e.g., regarding vaccinations, supporting children's physical and mental health) and credible, tailored resources that support decision making in clear, concise, plain language formats.

This information is critically important as we move beyond the pandemic, given that COVID‐19 illness is still prevalent, vaccines continue to be available and encouraged, and implications of the pandemic and related public health measures on mental health will persist for some time. Effective knowledge mobilisation efforts are needed to address parents' concerns and questions that have arisen during and will extend beyond the COVID‐19 pandemic.

## AUTHOR CONTRIBUTIONS

**Lisa Hartling**: Conceptualisation; investigation; funding acquisition; writing—original draft; methodology; validation; visualisation; writing—review and editing; formal analysis; supervision. **Sarah A. Elliott**: Conceptualisation; investigation; funding acquisition; writing—original draft; methodology; validation; visualisation; writing—review and editing; formal analysis; project administration; supervision; data curation. **Kelsey S. Wright**: Formal analysis; project administration; data curation; writing—original draft; methodology. **Lisa Knisley**: Conceptualisation; funding acquisition; investigation; methodology; writing—review and editing; project administration. **Shannon D. Scott**: Conceptualisation; investigation; funding acquisition; resources; methodology; writing—review and editing; formal analysis; project administration.

## CONFLICT OF INTEREST STATEMENT

The authors declare no conflict of interest.

## ETHICS STATEMENT

Ethics approval was obtained from the University of Alberta Health Research Ethics Board (Pro00112743) and all participants provided informed consent before participating in the study.

## Supporting information

Supporting information.

## Data Availability

As the data may allow identification of the focus group members, they are not publicly available. However, interested parties may contact the authors to discuss data sharing.
