# Supplementary material for: ‘It's quite a balancing act’: A qualitative study of parents' experiences and information needs related to the COVID‐19 pandemic
Source: Health Expect. 2024 Feb 22;27(1):e13994. doi: 10.1111/hex.13994 (PMC10884360; doi:10.1111/hex.13994)
Supplement: Supplementary file 1 — Supporting information. [file HEX-27-e13994-s001.docx]

# Appendix A: Semi Structured Interview Guide

1. Tell me about your experiences as a parent during the COVID19 pandemic.
   1. What were your concerns at the beginning of the pandemic?
   2. How did your experiences change throughout the pandemic?
   3. Did anyone in your family have COVID?
2. Tell me about your child’s experiences through the pandemic.
   1. What were your child’s concerns during this time?
   2. How did you address your child’s concerns?
3. What are your current concerns about COVID19?
   1. Do you have any concerns about your children being back in school or daycare?
   2. What are your experiences with public health restrictions in extracurricular activities for children?
   3. Do you have any concerns about the long-term effects of COVID?
4. What are your thoughts about the public health measures to prevent the spread of COVID19?
   1. What are your opinions on social distancing?
   2. What are your opinions of masks mandates?
   3. Are there any other measures or restrictions you have encountered?
5. How do your perspectives of public health measures align with your friends, family, and community members?
   1. How do you handle conflicting perspectives in your social circle?
6. Where do you seek information about the COVID19 virus?
   1. How did you evaluate the accuracy of the information?
   2. Did you experience any conflicting information about the COVID19 virus?
7. What supports and information would be helpful for you in your decisions related to COVID19?
